# Bilateral Nocardia farcinica Chorioretinitis With Endogenous Endophthalmitis and Concurrent CNS Disease: A Case Report

**DOI:** 10.7759/cureus.100697

**Published:** 2026-01-03

**Authors:** Rawan Ebada, Vaishnavi Balendiran, Ahmed Elkeeb

**Affiliations:** 1 Ophthalmology, University of Missouri School of Medicine, Columbia, USA

**Keywords:** chorioretinal lesions, disseminated nocardiosis, endogenous endophthalmitis, nocardia chorioretinitis, nocardia farcinica

## Abstract

Disseminated nocardiosis with endogenous endophthalmitis is a rare but life-threatening condition associated with significant morbidity and risk of permanent vision loss. We report a fatal case of bilateral chorioretinitis with unilateral endogenous endophthalmitis accompanied by soft tissue and central nervous system involvement caused by *Nocardia farcinica*. An 86-year-old woman presented with a five-day history of a progressively worsening blind spot in the left eye, and ophthalmic examination revealed bilateral chorioretinal lesions with marked anterior chamber and vitreous inflammation in the left eye. Soft tissue cultures grew *N. farcinica*, and subsequent imaging identified additional infectious foci in the lungs as well as the frontal and parietal lobes of the brain. Although an uncommon cause of endogenous endophthalmitis, *Nocardia* species, particularly the more resistant *N. farcinica*, should be considered in the differential diagnosis of intraocular infections, especially in immunocompromised patients, as bilateral ocular involvement and concomitant cerebral disease can pose substantial diagnostic and therapeutic challenges.

## Introduction

*Nocardia *spp are Gram-positive, obligate aerobic, weakly acid-fast filamentous bacteria commonly found in soil, water, and decaying matter [1]. Known to cause opportunistic infections, these organisms are more commonly seen in infections of the skin, soft tissues, and upper and lower respiratory tract. On rare occasions when ocular involvement is noted, Nocardia can cause conjunctivitis, keratitis, scleritis, pre-septal cellulitis, orbital cellulitis, dacryocystitis, and endophthalmitis [1-2]. There is evidence of increasing antimicrobial resistance of *Nocardia *spp, including *Nocardia farcinica* [3], which makes early identification and treatment, especially of advanced disease, even more important.

## Case presentation

An 86-year-old female presented with a five-day history of a black spot in her left eye that had progressed to involve her entire vision. Past medical history was significant for type 2 diabetes mellitus with nephropathy, coronary artery disease status post stenting of three vessels, Sjogren’s disease, and interstitial lung disease requiring 3L of oxygen by nasal cannula at baseline. Her home medications included hydroxychloroquine at 200mg/day and a taper of oral prednisone over the past several months, taking 10mg/day at the time of presentation. Her past ocular history was notable only for uncomplicated cataract surgery in both eyes six years prior to this presentation, as well as chronic dry eye syndrome in both eyes.

**Figure 1:**
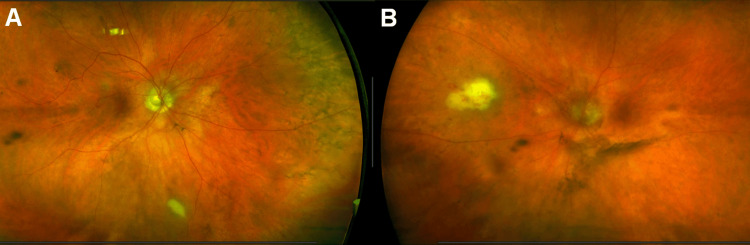
Fundus photos at presentation Ultra-widefield fundus images of the right (A) and left (B) eyes demonstrating bilateral white chorioretinal lesions with associated retinal hemorrhages and an overlying vitritis in the left eye.

Visual acuity (VA) was 20/30 in the right eye and 20/70 in the left eye. Slit lamp examination was unremarkable in the right eye and showed moderate inflammation of the anterior chamber and vitreous in the left eye. Interestingly, despite no symptomatic complaint from the patient and no evidence of inflammation on anterior segment exam, fundoscopy of the right eye (Figure 1A) showed a white chorioretinal lesion in the inferior periphery approximately 0.75 disc diameters (DD) in size. Posterior segment examination of the symptomatic left eye revealed a hazy media and a similar white chorioretinal lesion 2DD in size with overlying intraretinal hemorrhages in the nasal midperiphery (Figure 1B).

**Figure 2:**
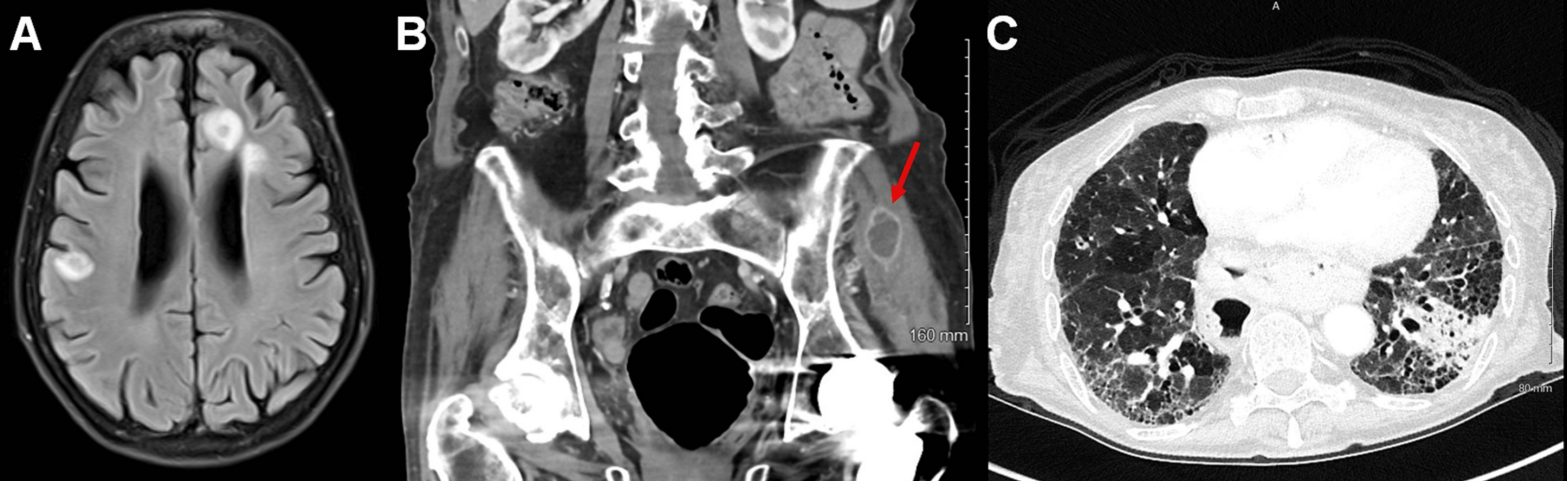
Extraocular imaging at presentation Imaging studies performed on day of presentation. A. MRI brain with and without contrast shows multiple ring-enhancing lesions of the bilateral cerebral hemispheres. B. CT abdomen with and without contrast demonstrates a ring-enhancing lesion of the left gluteus muscle (arrow). C. CT chest with and without contrast reveals a new left lower lobe consolidation concerning for pneumonia. There is a background honeycomb pattern bilaterally, consistent with the patient’s previously diagnosed interstitial lung disease.

Endogenous endophthalmitis of the left eye was immediately suspected and the patient admitted for systemic work-up. Magnetic resonance imaging (MRI) of the brain revealed six ring-enhancing lesions, some of which with vasogenic edema, in bilateral cerebral hemispheres and midbrain (Figure 2A). Further computed tomography (CT) imaging revealed ring-enhancing lesions of the left gluteus and right paraspinal muscles (Figure 2B) as well as a new focal left lower lobe consolidation concerning for pneumonia (Figure 2C). She was immediately started on broad-spectrum intravenous antibiotics and underwent aspiration of the left gluteal muscle abscess the next day. Gram stain and culture showed branching Gram-positive rods and partially acid-fast bacilli consistent with Nocardia species, and antibiotics were promptly narrowed to meropenem and trimethoprim/sulfamethoxazole (TMP/SMX) under advisement of the Infectious Disease service. After about a week, TMP/SMX had to be discontinued due to renal toxicity, and doxycycline was initiated. All other blood cultures, echocardiography, and infectious work-up were negative.

**Figure 3:**
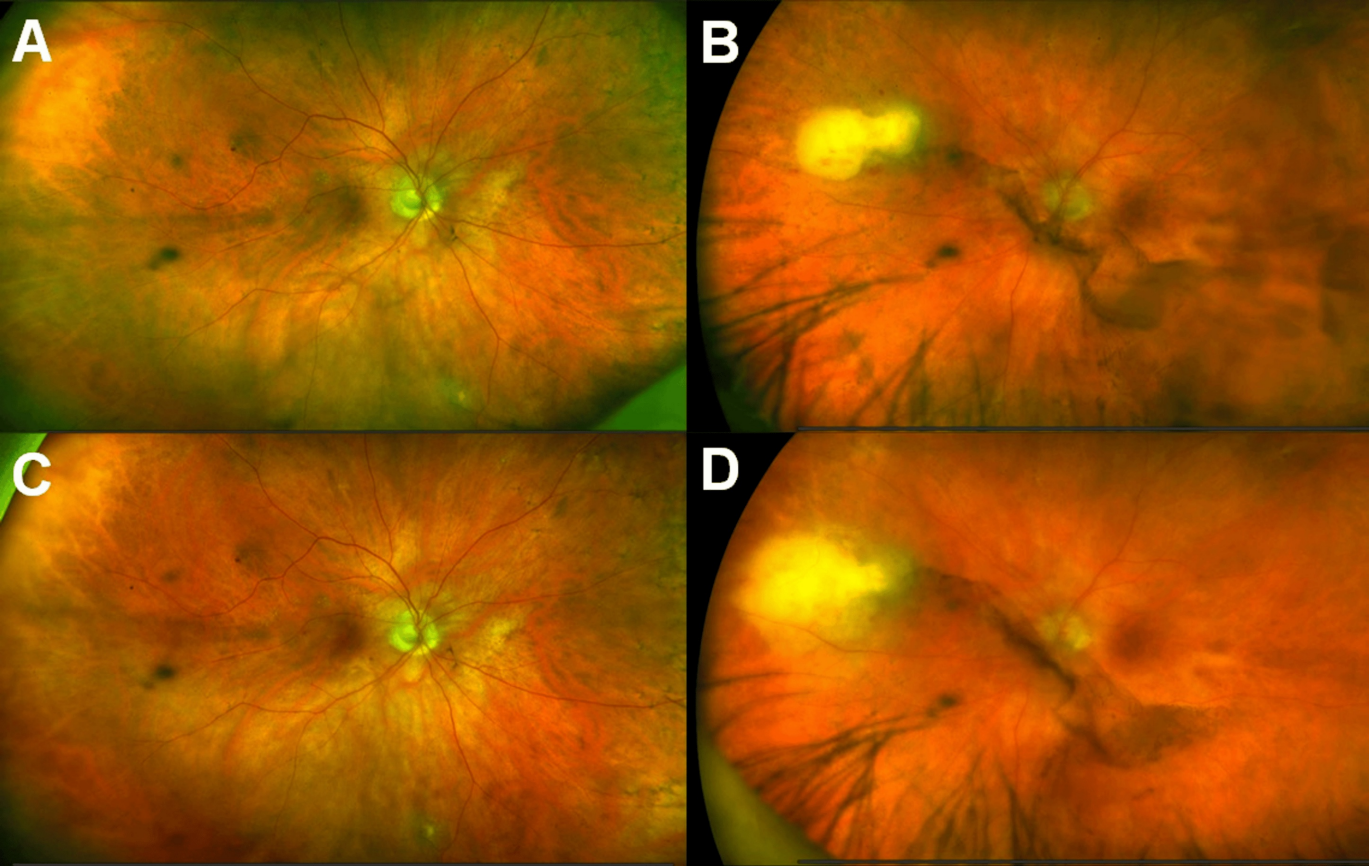
Fundus photos throughout inpatient course Ultra-widefield fundus images taken at two weeks (A, B) and three weeks (C, D) following presentation of the right (A, C) and left (B, D) eyes. The white chorioretinal lesion of the right eye shows improvement. However, the chorioretinal lesion of the left eye has largened at the second week (B), signifying subretinal abscess formation. This elevation improved by the third week (D).

She had a repeat ophthalmic examination two weeks after admission, at which time, she had received 14 days of intravenous meropenem and six days of intravenous doxycycline. VA was 20/30 in the right eye and 20/40 in the left eye. Slit lamp examination remained unremarkable in the right eye, and inflammation of the anterior chamber and vitreous in the left eye had slightly improved. The chorioretinal lesion of the right eye (Figure 3A) had markedly decreased in size to approximately 0.25 DD. However, the chorioretinal lesion of the left eye (Figure 3B) had increased to 3DD in size with significant elevation, suggesting formation of a subretinal abscess. On the same day, identification of her gluteal abscess culture returned with *Nocardia farcinica*. The samples were sent to an outside lab for sensitivities. Due to her worsening ocular exam, her antibiotic regimen was switched to intravenous amikacin and imipenem, as well as oral fluconazole due to concern of a fungal pathogen. After one week, the subretinal abscess in the left eye appeared less elevated and more diffuse, suggesting improvement (Figure 3D), while the right eye was unchanged (Figure 3C). Her visual acuity was stable. At this point, the patient had been admitted for three weeks, and voiced fatigue over her lengthy hospital stay. It was decided that she would continue her antimicrobial regimen on an outpatient basis with close follow-up with infectious disease and ophthalmology. A peripherally inserted central catheter (PICC) line was placed, and she was discharged with plan for follow-up with the Retina service in one week.

**Figure 4:**
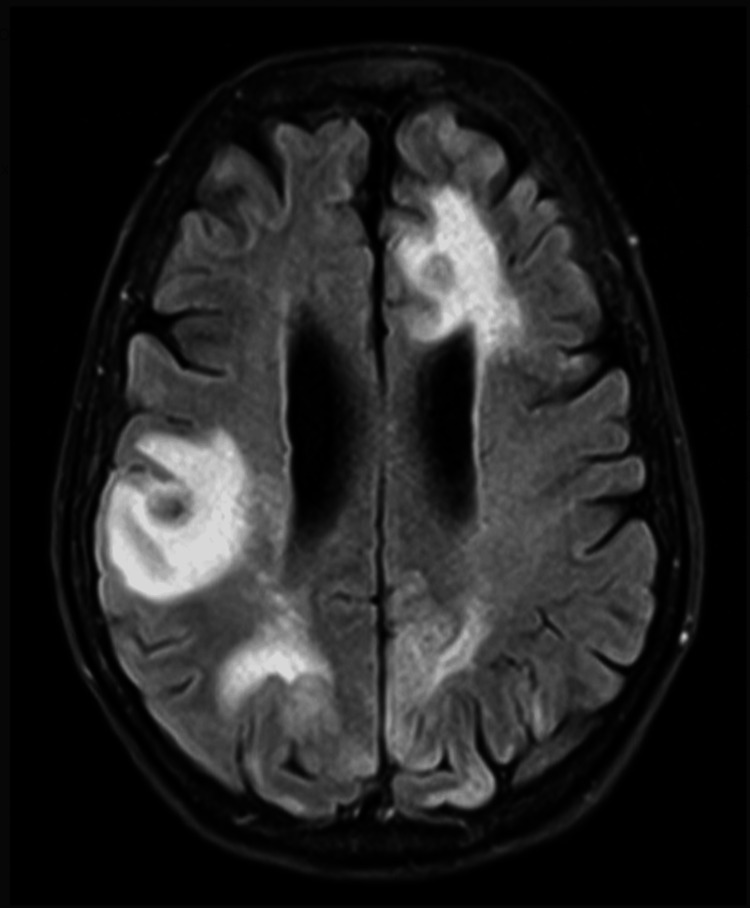
One-month neuroimaging MRI brain with and without contrast performed one month after initial presentation showing rim-enhancing lesions of the bilateral cerebral hemispheres as noted previous, but now also with associated leptomeningeal enhancement consistent with meningitis.

Unfortunately, six days following discharge, the patient returned to the hospital via ambulance for unresponsiveness. Her Glasgow Coma Scale (GCS) was 7 on arrival and further deteriorated to 4, prompting intubation for airway protection. MRI brain showed leptomeningeal enhancement consistent with associated meningitis (Figure 4), and she was found to be in focal status epilepticus on electroencephalogram (EEG). Discussion regarding her poor prognosis was held with her family, who opted for palliative care. She was terminally extubated and ultimately expired one month from her initial presentation to the eye clinic.

## Discussion

Endophthalmitis secondary to Nocardia is typically found in immunocompromised patients [4]; however, up to one-third of cases can occur in the immunocompetent [5]. While most ocular Nocardia infections are from an exogenous source, secondary to surgery or trauma, up to 12.5% arise from an endogenous source [1-5]. Case reports in the literature describe the occurrence of endogenous Nocardia endophthalmitis in patients with a history of granulomatosis with polyangiitis [4], myelodysplastic syndrome [5], nephrotic syndrome [6], status post liver transplant [7], and on chronic steroids for management of autoimmune hemolytic anemia [8] or membranoproliferative glomerulonephritis [9]. Although it typically arises from pulmonary infection, other extraocular sources of Nocardiosis include the skin and soft tissue as well as the central nervous system (CNS). The CNS is the most common extrapulmonary site, often with brain abscess formation, particularly in the setting of systemic steroids [4].

The ocular findings often include a creamy-white chorioretinal lesion with surrounding hemorrhage and retinal thickening with an overlying vitritis [5]. There may also be a mild anterior chamber reaction [6]. These findings are typically unilateral, although there are two cases of bilateral endophthalmitis from *N. asteroides* [9-10], one case from *N. farcinica* [11], and one case with concurrent *N. farcinica* and *N. kroppenstedtii* bacteremia [12] reported in the literature. The fluffy nature of the subretinal exudates often leads to the misdiagnosis of fungal endophthalmitis [1], and the prolonged incubation period of Nocardia species may further delay diagnosis and appropriate management, contributing to poor outcomes [8].

Although *Nocardia asteroides* remains the most commonly identified species in nocardial infections, *N. farcinica* accounts for a smaller but clinically significant proportion and is associated with increased virulence and a greater propensity for invasive disease. *N. farcinica* accounts for about 14% of infections and is more virulent with a higher propensity for invasive disease [3]. Importantly, *N. farcinica* demonstrates higher resistance to trimethoprim-sulfamethoxazole, a commonly used first-line agent, while susceptibility testing consistently shows preserved activity of amikacin and linezolid. Awareness of species-specific resistance patterns is therefore relevant when selecting empiric systemic therapy in severe or disseminated disease.

Due to the limited number of case reports on endogenous Nocardia endophthalmitis, there is no consensus on management strategy. Resolution of the infection and even desirable visual outcomes have been achieved with a variety of approaches, from serial intravitreal amikacin injections followed by a pars plana vitrectomy with membrane peel of a tractional membrane three months after the acute infection [7] to a combination of systemic and topical antimicrobials alone [8]. The common theme is that delayed diagnosis and concurrent CNS involvement suggest a poor prognosis in terms of mortality and ocular morbidity.

At time of presentation, this patient had already developed bilateral posterior uveitis in addition to multi-organ involvement of the soft tissue, lungs and CNS, marking an advanced degree of dissemination. As a result, our treatment strategy consisted mainly of systemic antimicrobial coverage. After weighing the patient’s excellent visual acuity and peripheral locations of the chorioretinal lesions against the potential risk of macular ischemia from intravitreal amikacin or the myriad of risks associated with pars plana vitrectomy, further intraocular intervention was decided against. This patient’s fatal outcome was likely due to the advanced level of CNS disease. Therefore, more aggressive intraocular intervention would not have averted this poor outcome.

In this case, initiation of systemic amikacin occurred two weeks after presentation, following speciation and susceptibility results. The combination of advanced disseminated disease and delayed administration of an agent with reliable activity against *N. farcinica* likely contributed to the poor outcome. Including this case, the limited reports of bilateral chorioretinitis due to *N. farcinica* have uniformly involved CNS disease and have been associated with high mortality. Of the two prior cases in the literature of bilateral chorioretinitis secondary to *Nocardia farcinica*, one patient ultimately expired while the other was successfully treated with a combination of intravenous imipenem, TMP/SMX, and amikacin along with intravitreal amikacin injection [11-12]. With the added context of our fatal case, clinicians encountering a presentation of bilateral chorioretinitis, CNS disease, and culture- or biopsy-proven Nocardiosis should be wary of a potential *Nocardia farcinica* infection and the associated mortality. Therefore, we argue that systemic amikacin should be part of first-line therapy in these cases until culture and sensitivities return.

## Conclusions

Endogenous Nocardia endophthalmitis in this case functioned as a clinical indicator of disseminated infection rather than an isolated ocular process. Bilateral posterior segment involvement in conjunction with central nervous system lesions was consistent with systemic spread and coincided with clinical deterioration despite apparent improvement in intraocular inflammation. Although speciation ultimately identified *Nocardia farcinica*, the clinical course highlights how delays in initiating systemic therapy with dependable nocardial coverage may allow progression of extraocular disease. Early ophthalmic recognition should therefore prompt expedited systemic evaluation and treatment, as prognosis is poor once multisystem involvement is established.

## References

[1] Dave VP, Pathengay A, Sharma S, Naveen N, Basu S, Pappuru RR, Das T (2019). Diagnosis, clinical presentations, and outcomes of Nocardia endophthalmitis. Am J Ophthalmol.

[2] Davitt B, Gehrs K, Bowers T (1998). Endogenous Nocardia endophthalmitis. Retina.

[3] Tan YE, Chen SC, Halliday CL (2020). Antimicrobial susceptibility profiles and species distribution of medically relevant Nocardia species: results from a large tertiary laboratory in Australia. J Glob Antimicrob Resist.

[4] Aguirre LE, Zamora Gonzalez RA, Barreto-Coelho P, Yannuzzi NA, Taldone SN (2020). Endogenous endophthalmitis heralding central nervous system involvement by Nocardia farcinica. Cureus.

[5] Lally DR, Sharma DK, Shields CL, Malloy BC, Garg SJ (2014). Pulmonary Nocardiosis initially manifesting as endogenous endophthalmitis. Can J Ophthalmol.

[6] Xu H, Fu B, Xu L, Sun J (2018). Disseminated Nocardiosis with subretinal abscess in a patient with nephrotic syndrome: a case report. BMC Ophthalmol.

[7] Hojjatie SL, Salek SS, Pearce WA, Wells JR, Yeh S (2020). Treatment of presumed Nocardia endophthalmitis and subretinal abscess with serial intravitreal amikacin injections and pars plana vitrectomy. J Ophthalmic Inflamm Infect.

[8] Kawakami H, Sawada A, Mochizuki K, Takahashi K, Muto T, Ohkusu K (2010). Endogenous Nocardia farcinica endophthalmitis. Jpn J Ophthalmol.

[9] Trehan H, Kaushik J, Jain VK, Parihar JK, Avasthi A (2017). Endogenous Nocardial endophthalmitis in an immunosuppressed patient: a serious warning of an underlying life threatening and blinding disorder. J Ophthalmic Vis Res.

[10] Sher NA, Hill CW, Eifrig DE (1977). Bilateral intraocular Nocardia asteroides infection. Arch Ophthalmol.

[11] Puri S, Hadayer A, Breaux A, Barr CC (2018). Disseminated Nocardiosis with retinal abscess in a patient treated for bullous pemphigoid. Am J Ophthalmol Case Rep.

[12] Venkat AG, Baynes K, Lowder CY, Srivastava SK, Sharma S (2019). A case report of endogenous endophthalmitis in the setting of Nocardia kroppenstedtii infection. Ophthalmic Surg Lasers Imaging Retina.

